# Assessment of the acute and subacute toxicity of the aqueous extract of Moroccan *Ferula communis* fruit in a mouse model

**DOI:** 10.1016/j.jsps.2023.101701

**Published:** 2023-07-07

**Authors:** Ghizlane Nouioura, Meryem Tourabi, Adel Tahraoui, Karima El-yagoubi, Souad Maache, Hinde Elfatemi, Badiaa Lyoussi, El houssine Derwich

**Affiliations:** a Laboratory of Natural Substances, Pharmacology, Environment, Modeling, Health and Quality of Life (SNAMOPEQ), Faculty of Sciences Dhar El-Mehraz, Sidi Mohamed Ben Abdellah University, Fez 30 000, Morocco; b Department of Biology and Earth Sciences, Regional Center for Education Careers and Train-ing of Fez-Meknes, Fez 30000, Morocco; c Departments of Pathology, University Hospital Hassan II, 30050 Fez, Morocco; d Unity of GC/MS and GC, City of Innovation, Sidi Mohamed Ben Abdellah University, Fez, Morocco

**Keywords:** *Ferula communis* L., Aqueous extract, Acute toxicity, Subacute toxicity, Mice

## Abstract

*Ferula communis* L. is thought to possess a wide range of therapeutic qualities. This plant's safety is critical regarding its potential uses as a medicine. Using the techniques outlined in the OECD recommendations, the present study aimed to assess the acute and subacute toxicity profiles of *Ferula communis* aqueous extract (FC-Ext) in mice. In the acute study, the FC-Ext was administered to adult male and female Swiss albino mice through oral and intraperitoneal routes at doses of 0–4 g/kg. The general behavioral effects, mortality rates, and latency of mortality were evaluated for a period of 14 days. For the sub-acute dose study, the FC-Ext was administered orally to adult mice at doses of 125, 250, and 500 mg/kg on a daily basis for 28 days. Body weight and selected biochemical and hematological parameters were measured, and histological examinations of the liver, kidney, and spleen were conducted to assess any signs of organ damage at the end of the treatment period. The results of the acute toxicity study demonstrated that the LD_50_ values for the oral and intraperitoneal administration of FC-Ext were 3.6 g/kg and 2.3 g/kg, respectively. In the subacute toxicity study of FC-Ext, no significant changes in body weight were observed. However, a substantial increase in the weights of the liver, kidney, and spleen was observed in male mice. The administration of FC-Ext to mice at doses higher than 250 mg/kg resulted in a decrease in white blood cells and platelets in both sexes and a reduction in red blood cells and mean corpuscular hemoglobin concentration in males and hemoglobin in females. No changes in biochemical parameters were observed. Microscopic examination of vital organs such as the liver, kidney, and spleen revealed no significant injuries. Based on the current results, the aqueous extract of *Ferula communis* has low toxicity. These findings provide important information about the toxicity profile of the traditional medicine plant *Ferula communis*.

## Graphical abstract

**Figure:**
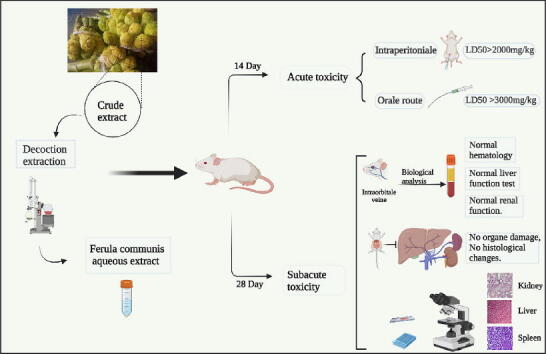

## 1 Introduction

Since ancient times, plants have been used for therapeutic purposes in numerous indigenous medical systems. Currently, herbal medication is gaining popularity all over the world, particularly in developing countries where medicinal plants are readily available and affordable. Additionally, people feel that natural medicines have less adverse effects than synthetic ones. However, the widespread perception that herbal medications are extremely safe and free of side effects is not only wrong, but also deceptive. In fact, several studies have found that therapeutic plants can cause a variety of unfavorable and harmful effects (Kharchoufa et al., 2018). Actually, it seems that the use of medicinal plants without checking for their toxicity poses a health hazard (Bellakhdar, 2004). It is crucial to study the harmful effects that can be induced by herbal remedies.

Acute and subacute toxicity tests are used to assess the potential harm and safety of chemicals. These tests involve evaluating the effects of single or repeated doses of a chemical on animals, and are often used to identify potential hazards and manage risks related to the production, handling, and use of chemicals (Y. Li et al., 2020). Acute toxicity tests involve administering a single dose of a chemical to assess the severity of toxic effects on the animal, while subacute toxicity tests involve administering repeated doses of a chemical to assess the potential for long-term effects on target tissues or organs. These tests can provide important information for hazard identification and risk management (Benrahou et al., 2022).

*Ferula communis* L. (Locally known as “Lbubale”, “Fassugh” or “Ouffale”) is an abundant, latex-containing perennial, glabrous, developed plant (from 1.5 to 3 m high). Due to its widespread distribution across the country, it is one of the distinctive taxa of the Moroccan flora (Bellakhdar, 1997). Infusions of the fruit and the aerial parts from *Ferula communis* are commonly used in Moroccan traditional medicine for their spasmolytic properties (Redouan et al., 2020; Mamoci et al., 2011). The rhizomes of this plant are used for skin diseases and hair care (Bellakhdar, 1978) and for sterility and weight gain (Ouarghidi et al., 2013) while the roasted flower buds are used to relieve dysentery and as antipyretic (Al-Yahya et al., 1998), as anthelmintic (Tavassoli et al., 2018) and as antidote-poison. Moreover, the resin of *Ferula communis* is commonly used Morocco as hypoglycemic and ritual and magic (Bnouham et al., 2010, Teixidor-Toneu et al., 2016).

The diverse pharmacological activities associated with the plant are attributed to a range of bioactive compounds predominantly extracted from its roots, leaves, and rhizomes. Notably, two different chemotypes of the plant are present, and each has a different set of effects. The toxic chemotype contains a significant amount of ferulenol, a prenylated coumarin compound that can induce ferulosis, a lethal hemorrhagic syndrome in animals and humans (Eruçar et al., 2023). On the other hand, the non-toxic chemotype displays a chromatographic profile characterized by a high concentration of sesquiterpenes and their derivatives, including ferutin, lapiferin, and teferin. Notably, the considerable presence of ferutin (ferutinol p-idroxybenzoate) predominantly accounts for the observed effects following the ingestion of Ferula extract (Sağıroğlu & Duman, 2010). Ferutin, a sesquiterpene, is derived from the plant's roots, leaves, and rhizome (Maiuolo et al., 2023). Moreover, Iranshahi et al. (2018) have identified various mechanisms through which compounds from different Ferula species inhibit cell growth.

To our knowledge, despite the widespread use of *Ferula communis* in folk medicine, no studies have been conducted to assess the toxicity of the fruit of this plant. Hence, the present study aimed to determine the acute and subacute oral toxicity of the aqueous extract from the fruit *Ferula communis* (FC-Ext) in mice with the hope that the findings would provide information about the safety of this extract in humans.

## 2 Material and methods

### 2.1 Plant material

The *Ferula communis* fruits were collected in the rural-commune Al Kasba Dar Al Hamra, Sefrou area (33° 41′ 45″ Nord, 4° 22′ 18″ Ouest), Morocco, between February and April 2022. The plant’s identity was confirmed by Dr. Amina Bari (from the Laboratory of Biotechnology and Conservation of Natural Resources, Faculty of Science, Sidi Mohamed Ben Abdellah University, Fez) and a voucher specimen (FC0522) was kept in the Herbarium of the same faculty. The fruits of *Ferula communis* were mechanically fragmented for later storage in sealed container bags at 0 °C after being dried at ambient temperature for seven days in an airy environment away from light.

### 2.2 Preparation of plant extract

An aqueous extract of the plant was prepared by boiling the dried powdered fruits of *Ferula communis* (50 g) in 500 ml distilled water under reflux for 20 min. The decoction obtained was centrifuged, filtered, and concentrated with vacuum to yield approximately 21% (w/w) of the residue, which was stored at 20 ◦C until used. The residue was dissolved in distilled water (FC-Ext) prior to the experiment every day.

### 2.3 Quantification of total phenolic content (TPC)

According to Singleton et al. (1999), the Folin-Ciocalteu reagent-based colorimetric method was used to quantify the total phenolic content (TPC). First, 450 μL of the Folin-Ciocalteu reagent solution (10%) was combined with 50 μL of gallic acid extract or a reference solution. After shaking on a vortex mixer, the mixture underwent a 5-minute incubation period. Following that, 450 μL of a 75 g.L^-1^ Na_2_CO_3_ solution was added, and the mixture was agitated one more. Using a spectrophotometer, the absorbance was measured at 760 nm after incubation for two hours at room temperature (25 ± 1C). For quantification, the standard curve equation with the gallic acid calibration curve (R^2^ = 0.9994) was applied. The outcomes are presented as the equivalent gallic acid (GAE) concentration per gram of dry plant material.

### 2.4 Quantification of total Flavonoids content (TFC)

To quantify the TFC, the sample of FC-Ext or quercetin (used as the positive control) were mixed with 150 μL of a 10% AlCl_3_ solution and 50 μL of a 5% sodium nitrite solution drawing from the comprehensive description previously provided by Nouioura et al. (2023). After an interval of six minutes, 200 μL of a 1% NaOH solution was added, and the mixture was thoroughly blended. The UV/Vis spectrophotometer evaluated the reagent mixture's absorbance at 510 nm following an hour of incubation at room temperature. The results were represented as milligrams of quercetin equivalent (QE) per gram of the sample (mg QE/g), with quercetin used to produce the standard curve in the concentration range of 0.003–0.5 mg/L (R^2^ = 0.9998). The tests were carried out in triplicate.

### 2.5 Antioxidant activity

(a) DPPH Free Radical Scavenging Activity

The total free radical scavenging capacity of *Ferula communis* fruit aqueous extract was estimated according to the previously reported method by Brand-Williams et al. (1995). For that, 50 µL of each extract of different concentrations (12.5 to 50 mg/ml) was added to 825 μL of an ethanol solution of DPPH. The mixture was vigorously shaken and incubated at room temperature for 1 h in the dark. The absorbance of the DPPH radical in the blank (without antioxidants) was also measured. All determinations were performed in triplicate. The ability to scavenge DPPH radicals was calculated using the following equation:

(1) $$Inhibition\;\left(\%\right)=\frac{Abs\;control-Abs\;sample}{Abs\;control}\times 100$$

Where:

Abs control: Absorbance of the control.

Abs sample: Absorbance of the extract.

The IC_50_ value of DPPH was determined by analyzing the graph depicting the percentage of inhibition exhibited by the plant extract.

(b) ABTS radical scavenging activity

As described by Re et al. (Re et al., 1999), the free radical scavenging activity of wild *Ferula* fruit extracts was determined by an ABTS free radical cation decolorization assay. In a nutshell, 50 μL of different dilutions of each aqueous extract or gallic acid (used as positive control) were added to 825 μL of ABTS radical cation solution. The solutions were incubated in the dark for 6 min at room temperature. The absorption measurements were made at 734 nm by UV/Vis spectrophotometer. The absorption of a blank sample containing the same amount of ethanol and ABTS solution served as a negative control. The percent inhibition of absorbance was calculated using the formula (1), and IC_50_ values were determined graphically and expressed in mg/mL. All analyses were done in triplicate.

### 2.6 Animals used

Male and female Swiss Albino mice (25–35 g) were obtained from the animal house, Faculty of Science, Sidi Mohamed Ben Abdellah University, Fez. Mice were acclimatized in a room at a temperature of 25 ± 1 ◦C, with photoperiod of 12 h (12 L/12 D), and provided tap water and food ad-libitum. The procedures used to carry out this study were approved by our institutional committee on animal protection as per guidelines of the ethical approval registered under the number L.20. USMBA-SNAMOPEQ 2020–03.

### 2.7 Acute toxicity study of FC-Ext

The acute toxicity test was carried out in accordance with OECD (Organization for Economic Co-operation and Development) 423 (Test no. 423: acute oral toxicity-acute toxic class method, 2002). Two administration methods have been used: one oral, intended for human usage, and the other intraperitoneal, which ensures systemic exposure to the drug. The animals received a constant volume of test materials of the order of 0.5 ml/20 g body weight (BW) and 0.4 ml/20 g BW for oral and intraperitoneal route respectively (Tahraoui et al., 2010). The FC-Ext (dissolved in distilled water) was administered by gavage or by intraperitoneal route at doses of 0, 0.1, 0.5, 1.0, 1.5, 2, 2.5, 3, 3.5, 4 g/kg BW.

Following treatment, the animals were monitored continuously for 1 h, then intermittently for 4 h, and thereafter over a period of 24 h to look for behavioral modifications, toxicity and/or death symptoms, as well as the latency of death. For 14 days, Animals were provided food and water, and their daily food intake, water consumption, body weight, death and visual changes were all recorded. The LD_50_ values were determined according to the method of Litchfield and Wilcoxon (Litchfield Jr & Wilcoxon, 1949).

### 2.8 Sub-acute toxicity study of FC-Ext

Subacute toxicity study of the FC-Ext was conducted according to OECD’s recommendations (Guideline n°407). A total of 40 mice were split into four groups, each of which had both male and female animals (5 males and 5 females). Over a period of 28 days, Group (1) (control group) received 1 ml/100 g of distilled water, whereas Groups 2, 3, and 4 received orally FC-Ext at doses of 125 mg/Kg, 250 mg/Kg, and 500 mg/Kg of BW, respectively (Darbar et al., 2019).

### 2.9 Measurement of hematological and biochemical parameters

The refined *retro*-orbital bleeding method, utilizing the lateral approach described by Sharma et al. (2014), was employed to obtain blood samples of adequate volume and quality. This technique involves gently removing the eyelid and stretching the upper eyelid to expose the eye, allowing for careful retrieval of blood using a Pasteur pipette with minimal vein irritation. After collection, the blood samples are divided into two portions. One half is deposited into an EDTA aliquot tube for hematological assays, while the other undergoes centrifugation. The centrifuged samples are further processed to isolate the serum intended for future biochemical analysis.

The Sysmex KX-21 (Sysmex Corp., Japan), an automated blood analyzer, was utilized to determine a comprehensive panel of hematological parameters, including total red blood cell (RBC) count, hemoglobin (HGB) level, mean RBC hemoglobin concentration (MCHC), hematocrit (HCT), white blood cell (WBC) count, platelet (PLT) count, and WBC differential.

For the biochemical analyses, serum samples were collected and analyzed for liver enzymes such as aspartate aminotransferases (AST) and alanine aminotransferases (ALT). Additionally, serum kidney parameters including urea and creatinine were quantified using an automated serum biochemistry analyzer from Beckman Coulter (Japan).

### 2.10 Histopathology evaluation

Following a simple laparotomy, the liver, kidneys, and spleen were meticulously extracted in a sterile environment to facilitate macroscopic examination and determination of relative weights. Subsequently, the organs were promptly preserved in 10% buffered formalin (pH 7.4). Once fixed, the tissue samples underwent dehydration through a graded series of ethanol (70–100%) and clearance with toluene. They were then embedded in paraffin for subsequent sectioning using a microtome, resulting in thin sections measuring 5 µm. These sections were subjected to Haematoxylin and Eosin (HE) staining to enable subsequent microscopic analysis.

### 2.11 Statistical analysis

Data were entered in Excel file (Microsoft Office 2016) and analyzed with GraphPad Prism version 8.0 for Windows software. All results are expressed as mean value ± standard error of the mean (S.E.M.). One-way ANOVA followed by post hoc Tukey's test were used to statistically compare the groups. Results were considered significant at p < 0.05.

## 3 Results

### 3.1 Quantification of TCP, TFC and antioxidant activity

In the obtained aqueous extract from the fruit of *Ferula communis* (FC-Ext), the quantification results for phenolic and flavonoid content and antioxidant activity are as follows: Total phenolic content (TCP) was found to be 44.04 ± 0.22 mg GAE/g DW, whereas total flavonoid content (TFC) was found to be 8.97 ± 0.47 mg QE/g DW. These numbers provide insightful information regarding the concentration of phenolic and flavonoid in FC-Ext. In terms of antioxidant activity, it was found that the IC_50_ DPPH and IC_50_ ABTS levels of the FC-Ext were 42.15 ± 0.06 µg/mL and 2.3 ± 0.02 µg/mL, respectively.

### 3.2 Acute toxicity

No signs of toxicity or deaths were observed after oral administration of the FC-Ext up to the dose of 1.5 g/kg BW, which is the no-observed-adverse-effect level (NOAEL) for both sexes (Dorato & Engelhardt, 2005). Regarding the doses ranging from 2 to 3.5 g/kg BW, adverse effects, such as anorexia, ataxia, hypoactivity, piloerection, and syncope with a mortality rate up to 60% were noticed mainly in female mice. Above the dose of 3.5 g/kg BW, which was the lowest-observed-adverse-effect level (LOAEL) for the males, we noticed that signs of toxicity and motility begun to appear in males, along with an upsurge in female symptoms and mortality. The overall calculated acute toxicity (LD_50_) of oral administered FC-Ext in mice was 3.6 g/kg BW (Table 1).

**Table 1:** Acute toxicity study of the aqueous extract of *Ferula communis* administered orally and intraperitoneally in mice.

**Dose (g/kg BW)**	**Sex**	**Administration route**					
		**Oral**			**Intraperitoneal**		
		**D/T**	**Latency**	**Toxic symptoms**	**D/T**	**Latency**	**Toxic symptoms**
**Control**	Male	0/5	–	None	0/5	–	None
	Female	0/5	–	None	0/5	–	None
**0.1**	Male	0/5	–	None	0/5	–	None
	Female	0/5	–	None	0/5	–	None
**0.5**	Male	0/5	–	None	0/5	–	None
	Female	0/5	–	None	0/5	–	None
**1.0**	Male	0/5	–	None	0/5	–	None
	Female	0/5	–	None	0/5	–	None
**1.5**	Male	0/5	–	None	1/5	> 48 h, < 72 h	Ataxia, hypoactivity,
	Female	0/5	–	None	1/5	> 48 h, < 72 h	Ataxia, hypoactivity,
**2.0**	Male	0/5	–	None	2/5	> 48 h, < 72 h	Anorexia, Ataxia, hypoactivity, piloerection.
	Female	1/5	> 72 h, < 96 h	Anorexia, Ataxia, hypoactivity,piloerection	3/5	> 24 h, < 48 h	Anorexia, Ataxia, hypoactivity, piloerection.
**2.5**	Male	0/5	–	None	2/5	> 24 h, < 48 h	Anorexia, Ataxia, hypoactivity, piloerection, asthenia, convulsions.
	Female	1/5	> 72 h, < 96 h	Anorexia, Ataxia, hypoactivity, piloerection.	3/5	< 12 h	Anorexia, Ataxia, hypoactivity, piloerection, asthenia, convulsions.
**3**	Male	0/5	–	None	3/5	< 12 h	Anorexia, Ataxia, hypoactivity, piloerection, Lethargy, asthenia, asthenia, convulsions,
	Female	2/5	> 48 h, < 72 h	Anorexia, Ataxia, hypoactivity, syncope.	5/5	< 12 h	Anorexia, Ataxia, hypoactivity, piloerection, Lethargy, asthenia, convulsions, syncope.

**Dose (g/kg BW)**	**Sex**	**Administration route**					
		**Oral**			**Intraperitoneal**		
		**D/T**	**Latency**	**Symptoms**	**D/T**	**Latency**	**Symptoms**
**3.5**	Male	0/5	–	None	4/5	< 12h	Anorexia, Ataxia, hypoactivity, piloerection, Lethargy, asthenia, convulsions, syncope.
	Female	3/5	> 12h, < 24h	Anorexia, Ataxia, hypoactivity, syncope,	5/5	> 1h, < 2h	Anorexia, Ataxia, hypoactivity, Lethargy, asthenia, convulsions, syncope.
**4**	Male	2/5	> 12h, < 24h	diarrhea with stools and urine deeply yellow, atypical locomotion (back limbs falling), syncope.	5/5	> 1h, < 2h	Anorexia, Ataxia, hypoactivity, Lethargy, asthenia, convulsions, syncope.
	Female	5/5	> 12h, < 24h	Anorexia, Ataxia, hypoactivity, Lethargy, asthenia, convulsions	5/5	> 1h, < 2h	Anorexia, Ataxia, hypoactivity, Lethargy, asthenia, convulsions, syncope.

D/T = dead/treated mice; none = no toxic symptoms during the observation period; mortality latency = time to death (in hours) after the injection. Latency time elapsed between administration and death.

Additionally, for both sexes, the toxic symptoms (ataxia, hypoactivity, piloerection…) as well as the mortality rate of the intraperitoneally administered FC-Ext increased rapidly for the doses above the LOAEL (equal to 1 g/kg BW). The 4 g/kg BW dose of the extract resulted in the death of all animals. The calculated acute toxicity (LD_50_) of intraperitoneal administered FC-Ext in mice was 2.3 g/kg BW.

### 3.3 Subacute toxicity

a) Body weights

**Fig. 1:**
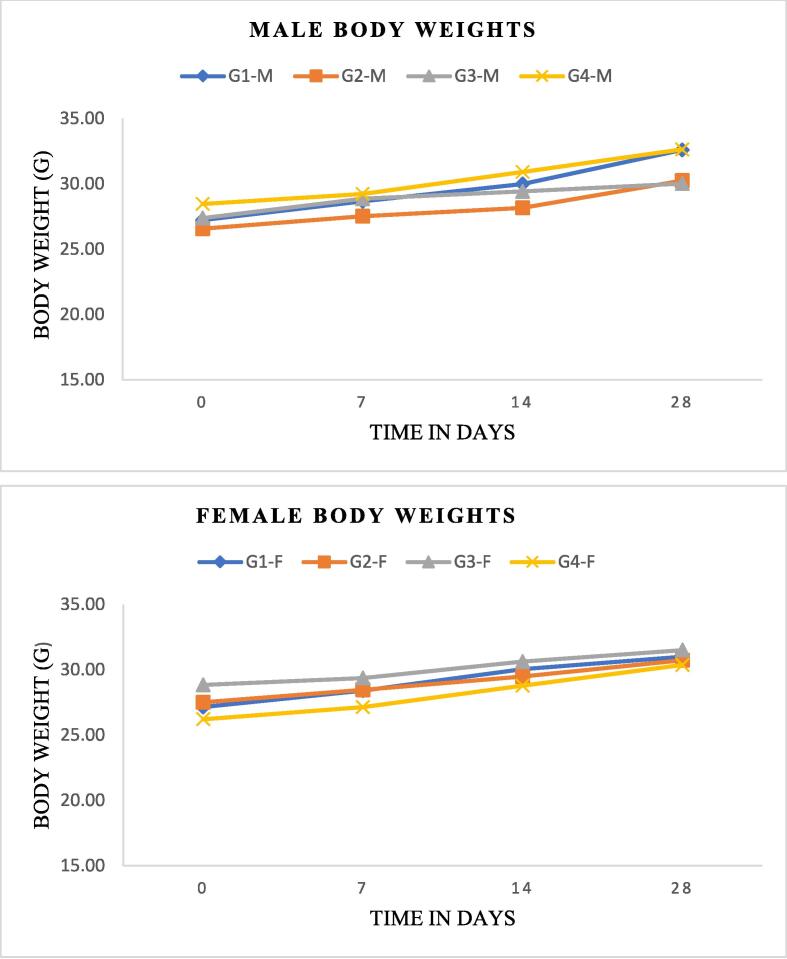
The effect of sub-acute oral treatment with the aqueous extract of *Ferula communis* on body weight in male and female mice. The aqueous FC-Ext was given orally to mice in four groups at the following doses for a period of 28 days: Group I (control, 0 mg/kg), Group II (FC dose, 120 mg/kg), Group III (FC dose, 250 mg/kg), and Group IV (FC dose, 500 mg/kg). The data are expressed as mean ± S.E.M.

Changes in body weight in mice with sub-chronic toxicity are illustrated in Fig. 1. In both males and females, no significant differences in body weight changes were noted between the control group (G1) and any of the treatment groups (G2-G4) at any time period (p > 0.005). In addition, during the course of the 28-day treatment, no lethality was observed at any dose up to a maximum of 500 mg/kg.

b) Evaluation of the organ weights

**Fig. 2:**
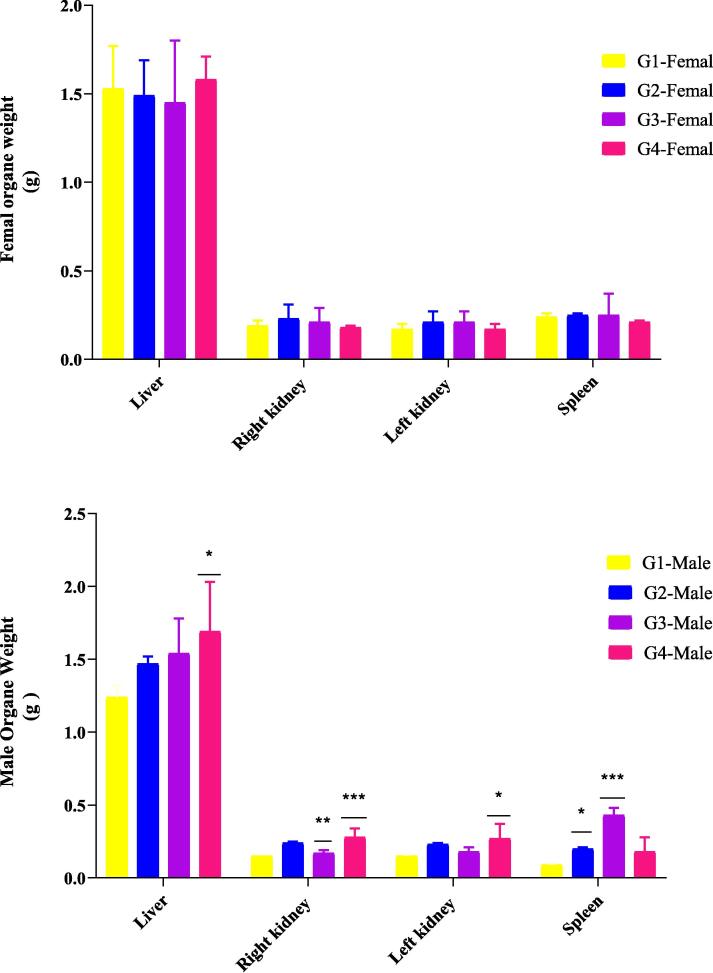
The effect of sub-acute oral treatment with the aqueous extract of *Ferula communis* on organ weight (liver, kidney, and spleen) in female and male mice. The aqueous FC-Ext was given orally to mice in four groups at the following doses for a period of 28 days: Group I (control, 0 mg/kg), Group II (FC dose, 120 mg/kg), Group III (FC dose, 250 mg/kg), and Group IV (FC dose, 500 mg/kg). The data are expressed as mean ± S.E.M. Significant differences in each group versus the controls were as follows: * p < 0.05, ** p < 0.01, *** p < 0.001.

The vital organs (liver, kidneys, and spleen) of male and female mice were weighed after 28 days of FC-Ext administration at doses of 125, 250, and 500 mg/kg. In male mice, the kidneys and liver of the group treated with 500 mg/kg showed a statistically significant increase in weight compared to the control group. The spleen also showed a statistically significant increase in weight, particularly in the group treated at 250 mg/kg. In female mice, there was no alteration in the weight of these organs compared to the control group (Fig. 2).

c) Hematological parameters

The effect of sub-chronic oral administration of FC-Ext on the hematological parameters is summarized in Table 2. In males, we noticed a significant decrease (*p* < 0.05) when compared with the control in red blood cell (RBC) count (–22.4%), The mean corpuscular hemoglobin concentration (MCHC) (-20%), white blood cell (WBC) Count (-53.9%), platelet count (-10% approx.) and differential count of eosinophils, neutrophils, and lymphocytes in the G4 group treated at the dose 500 mg/kg.

**Table 2:** The effect of sub-acute oral administration of the aqueous extract of *Ferula communis* on certain hematological parameters in mice.

	**Parameters**		**G I**	**G II**	**G III**	**G IV**
**Males**	RBC (10^6^ µl^−1^)		10.62 ± 0.67	10.08 ± 0.12	9.82 ± 1.32	8.24 ± 0.17*
	HGB (g dl^−1^)		12.70 ± 0.56	12.67 ± 0.15	10.45 ± 0.65	12.20 ± 3.32
	MCHC (g dl^−1^)		36.85 ± 0.78	29.15 ± 0.92***	29.70 ± 0.71***	31.10 ± 2.12***
	HCT (%)		44.57 ± 1.85	43.43 ± 0.64	38.67 ± 1.31	41.80 ± 13.61
	WBC (10^3^ µl^−1^)		12.47 ± 1.22	10.68 ± 0.59	11.46 ± 3.52	5.74 ± 2.84**
	PLT (10^3^ µl^−1^)		821.00 ± 5.45	856.00 ± 5.75	806.00 ± 7.15	740.00 ± 15.56**
	Differential count (%)	Eosinophils	2.20 ± 0.98	2.17 ± 0.40	1.67 ± 0.49	0.50 ± 0.5**
		Neutrophils	13.88 ± 1.31	14.27 ± 2.50	14.65 ± 1.27	10.20 ± 0.89*
		Lymphocytes	86.00 ± 2.12	84.93 ± 2.61	84.60 ± 2.51	90.53 ± 0.76*
		Monocytes	2.17 ± 1.00	2.23 ± 1.18	0.70 ± 0.36	2.57 ± 0.45
		Basophil	0.10 ± 0.01	0.14 ± 0.14	0.17 ± 0.15	0.17 ± 0.11
**Females**	RBC (10^6^ µl^−1^)		10.74 ± 1.13	10.93 ± 0.80	9.79 ± 0.09	9.73 ± 0.63
	HGB (g dl^−1^)		15.00 ± 0.79	14.37 ± 0.40	14.47 ± 2.00	12.40 ± 1.31*
	MCHC (g dl^−1^)		29.75 ± 0.92	29.55 ± 0.07	27.65 ± 3.32	30.00 ± 1.13
	HCT (%)		48.10 ± 1.51	43.03 ± 2.46	48.29 ± 7.39	48.00 ± 2.08
	WBC (10^3^ µl^−1^)		13.80 ± 0.64	13.13 ± 2.57	9.72 ± 0.29*	9.71 ± 1.55*
	PLT (103 µL − 1)		867.00 ± 9.02	865.00 ± 7.05	801.00 ± 9.31***	698.00 ± 6,12***
	Differential count (%)	Eosinophils	1.00 ± 0.98	2.04 ± 0.99	1.37 ± 0.49	0.30 ± 0.60
		Neutrophils	16.00 ± 3.40	15.80 ± 1.09	15.62 ± 0.98	10.20 ± 1.23*
		Lymphocytes	83.83 ± 2.06	81.80 ± 0.85	75.73 ± 1.74	76.87 ± 10.30
		Monocytes	2.10 ± 0.50	1.53 ± 0.51	1.33 ± 0.32*	0.80 ± 0.20*
		Basophil	0	0	0	0

The aqueous FC-Ext was administered orally to mice in four groups at the following doses for a period of 28 days: Group I (control, 0 mg/kg), Group II (FC dose, 120 mg/kg), Group III (FC dose, 250 mg/kg), and Group IV (FC dose, 500 mg/kg).

**HBG:** hemoglobin**, HCT**: hematocrit, **RBC:** red blood cells, **MCHC**: Mean corpuscular hemoglobin concentration, **PLT**: platelet, **WBC:** white blood cells.

The data are defined as mean ± S.E.M. Significant differences in each group versus the controls were as follows: * *p* < 0.05, ^**^p < 0.01, ^***^p < 0.001.

On the other hand, females treated with the dose of 500 mg/kg (G4) showed a significant reduction (p < 0.05), when compared to the control group, for hemoglobin (HGB) (-17.3%), WBC (-29.6%) and differential count of neutrophils and monocytes. It should be noted that Group 3 (receiving 250 mg/kg of FC-Ext) showed a significant decrease in WBC (-29.5%), platelet (-19%) and differential count of monocytes.

d) Serum biochemical parameters

Biochemical parameters, of the treated and control mice, such as aminotransferases (AST and ALT), urea, and creatinine are shown in Table 3. The present results outlined that repeated oral administration of FC-Ext (up to a daily dose of 500 mg/kg BW for 28 days) did not cause significant changes in plasma creatinine, urea and the liver marker enzymes (ALT and AST).

e) Histological analysis

**Table 3:** Effect of sub-acute oral administration of the aqueous extract of *Ferula communis* on some biochemical parameters in mice.

**Groups**	**Males**				**Females**			
	**G I**	**G II**	**G III**	**G IV**	**G I**	**G II**	**G III**	**G IV**
**AST (U/L)**	144.50 ± 33.32	135.00 ± 24.04	141.00 ± 15.55	136.00 ± 11.31	149.00 ± 21.21	131.00 ± 15.55	169.00 ± 15.55	170.50 ± 4.95
**ALT (U/L)**	35.50 ± 6.36	36.00 ± 0.00	44.00 ± 2.82	44.00 ± 24.04	40.00 ± 4.24	40.00 ± 16.97	35.00 ± 9.90	32.00 ± 1.41
**Urea (g/l)**	0.37 ± 0.08	0.28 ± 0.02	0.50 ± 0.05	0.29 ± 0.30	0.28 ± 0,01	0.45 ± 0.19	0.56 ± 0.38	0.28 ± 0.01
**Crea (mg/l)**	3.45 ± 0.21	3.90 ± 0.28	4.45 ± 0.77	2.90 ± 0.98	3.20 ± 0.42	4.60 ± 1.27	5.20 ± 1.90	3.50 ± 0.56

The aqueous FC-Ext was administered orally to mice in four groups at the following doses for a period of 28 days: Group I (control, 0 mg/kg), Group II (FC dose, 120 mg/kg), Group III (FC dose, 250 mg/kg), and Group IV (FC dose, 500 mg/kg).

The data are expressed as mean ± S.E.M.

**Fig. 3:**
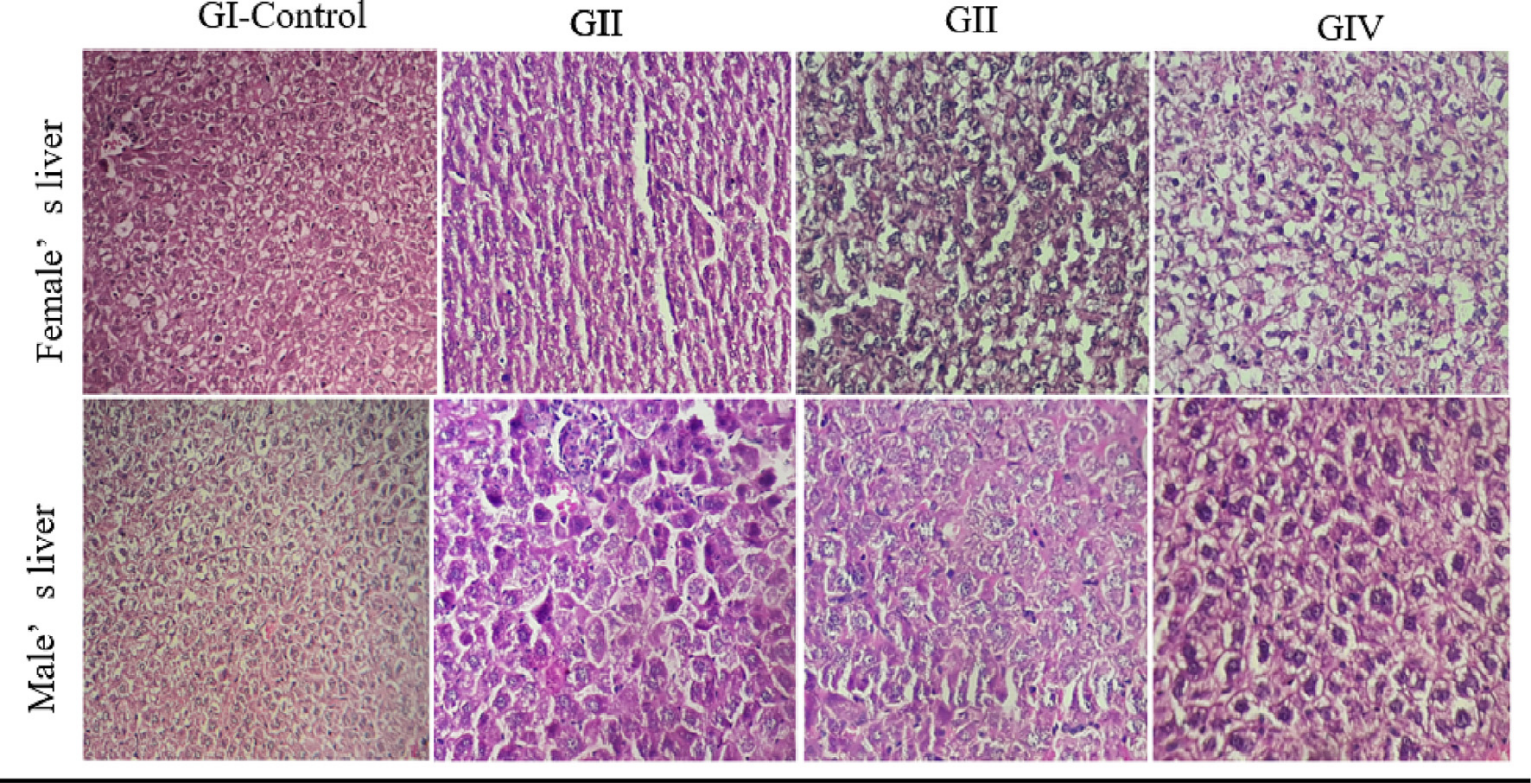
Histological examination of the liver in male and female mice following subacute treatment with the aqueous extract of *Ferula communis*. GI (control), GII (125 mg/kg bw), GIII (250 mg/kg bw), GIV (500 mg/kg bw). (HE × 40).

**Fig. 4:**
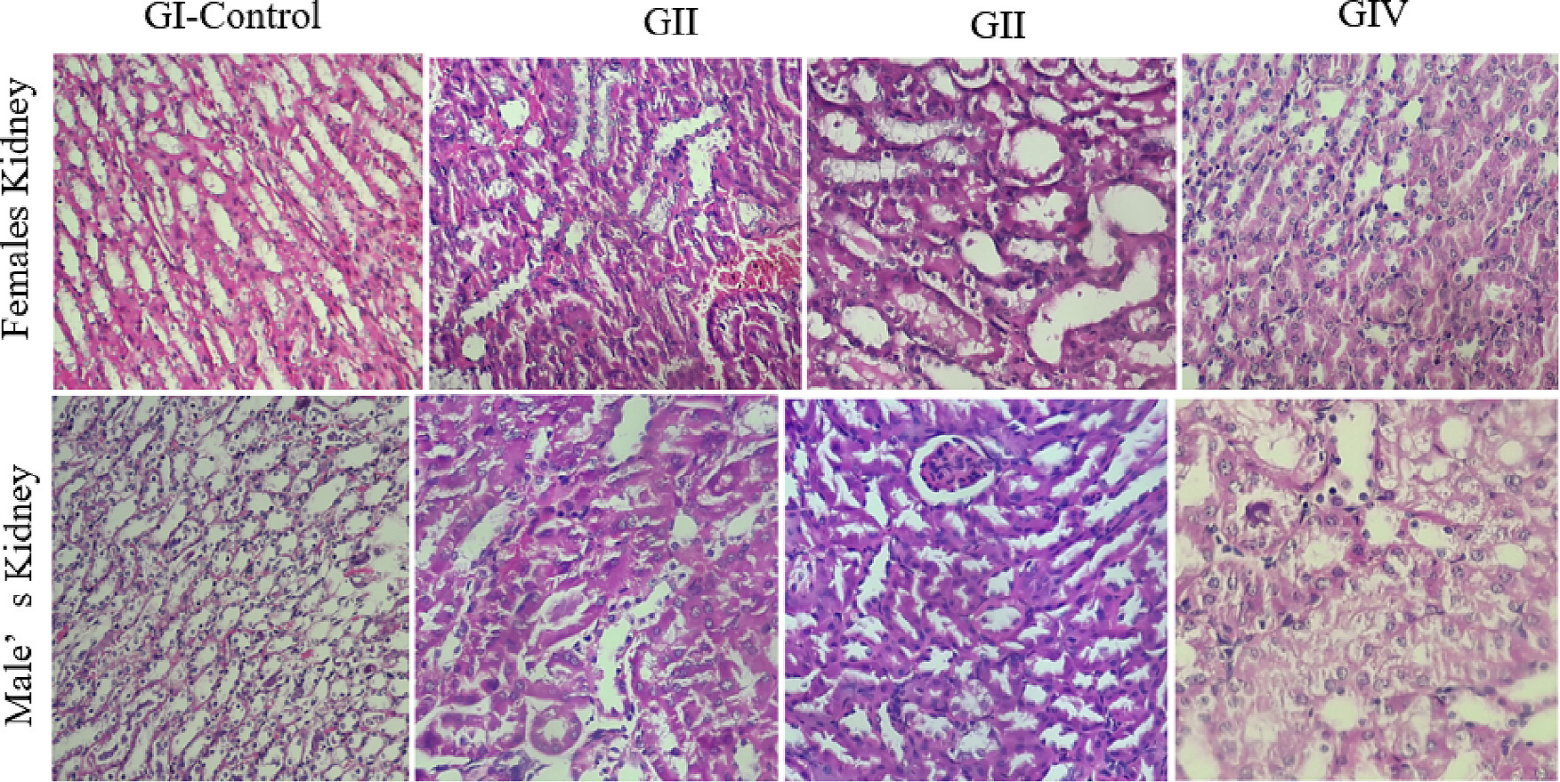
Histological examination of the kidney in male and female mice following subacute treatment with the aqueous extract of *Ferula communis*. GI (control), GII (125 mg/kg bw), GIII (250 mg/kg bw), GIV (500 mg/kg bw). (HE × 40).

**Fig. 5:**
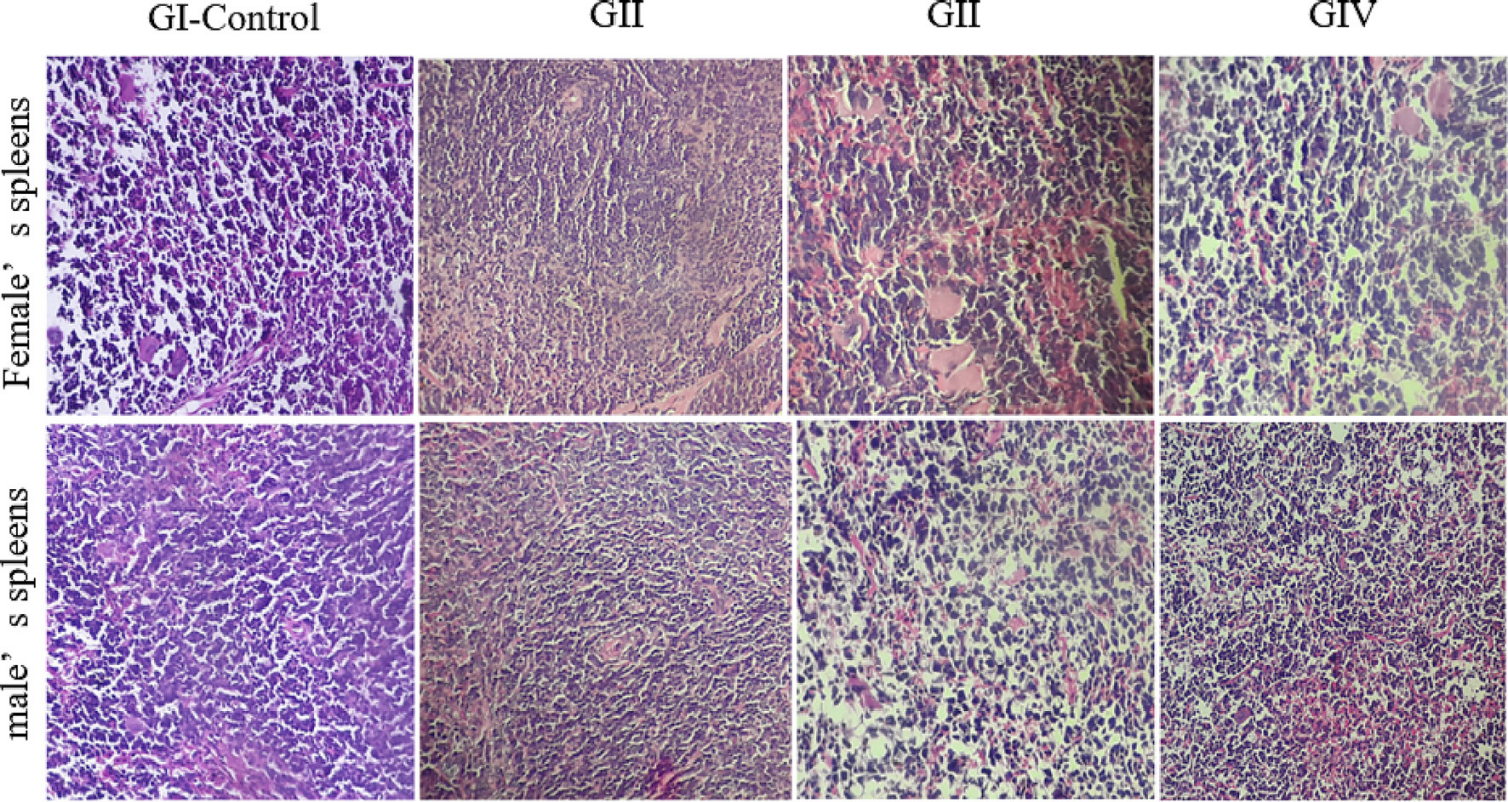
Histological examination of the spleen in male and female mice following subacute treatment with the aqueous extract of *Ferula communis*. GI (control), GII (125 mg/kg bw), GIII (250 mg/kg bw), GIV (500 mg/kg bw). (HE × 40).

The kidneys, liver, and spleen showed normal morphology and no macroscopic lesions were observed. Histopathological examination of these organs also confirmed the absence of anatomical abnormalities. Treatment with doses ranging from 125 to 500 mg/kg did not result in significant changes in the cellular architecture of these tissues in male and female mice (Fig. 3, Fig. 4, Fig. 5). These findings were supported by the biochemical parameters, which remained unchanged in terms of AST, ALT, and creatinine.

## 4 Discussion

*Ferula communis,* a plant native to the Mediterranean region, has a long history of medicinal use and is known for its various pharmaceutical activities due to the presence of bioactive compounds in the roots, leaves, and rhizome. The plant is divided into two chemotypes, each with distinct effects due to the presence of different compounds (Louvet et al., 2015). The toxic chemotype contains a high percentage of ferulenol, a prenylated coumarin compound that can cause ferulosis, a lethal hemorrhagic syndrome in animals and humans. The non-toxic chemotype of *F. communis* is traditionally used to treat a variety of conditions, including dysentery, skin diseases, and microbial and fungal infections (Macrì et al., 2020). In order to properly evaluate the potential toxicity of *F. communis*, which is commonly used in traditional medicine, both acute and subacute toxicity were studied.

The present study revealed that the FC-Ext exhibited a high concentration of phenolic compounds. These findings align with those of Gamal and Atraiki (2015), who utilized n-butanol and ethyl acetate extracts and reported a range of 44.7–55.8 mg gallic acid equivalent per gram of dry weight. In contrast, Rahali et al. (2019) reported a higher total phenolic content (TPC) for the Tunisian fruit extract, with a value of 422 mg GAE/g DW, followed by flowers (207.21 mg GAE/g DW) and stems (129.86 mg GAE/g DW). Regarding total flavonoid content (TFC), Rahali et al. (2019) investigated various aerial parts extracts of *Ferula communis* L. (flowers, fruits, and stems) using organic solvents. They found that the fruits and stems exhibited TFC values of 14.23 and 13.37 mg QE/g DW, respectively, while the methanolic extract of flowers had the highest TFC value of 48.77 mg QE/g DW. Considering the significant antioxidant potential observed in our study, it can be concluded that the FC-Ext demonstrated noteworthy free radical scavenging activity.

According to toxicity classification, substances with LD_50_ values within the range of 1–5 g/kg are generally considered to have low toxicity, while those with values higher than 5.0 g/kg are generally considered to be non-toxic (Piyachaturawat et al., 2002). The LD_50_ values for FC-Ext in mice were found to be within the range of 1–5 g/kg when administered orally or intraperitoneally. These results suggest that the aqueous extract of *Ferula communis* is practically low-toxic when administered through either of these routes. It is worth noting that the toxicity of FC-Ext was higher when it was administered intraperitoneally compared to orally. This higher toxicity of the extract through the intraperitoneal route may be due to the increased first pass effect and lower rate of absorption associated with oral treatment.

During the subacute tests, changes in body weight were used as an indicator of the potential adverse effects of the administered drugs. Both the treated and control groups showed a normal, progressive increase in mean body weight. The weight gain between the control group and the groups treated with the FC-Ext at a dose up to 500 mg/kg was not significantly different. This may be due, at least in part, to the fact that the FC-Ext did not negatively impact appetite or food intake. Previous research has indicated that organ weights can be a useful measure of the health and well-being of animals (Raina et al., 2015). It is possible that herbal products may be toxic to certain organs, such as the liver and kidneys, which play important roles in the animal body. However, the results of the present study did not reveal any significant changes in organ weight in the liver, kidney, or spleen of the treated groups compared to the control group.

Nutrients and foreign substances are primarily transported throughout the body via the bloodstream. Therefore, the blood's components (erythrocytes, leukocytes, platelets, and hemoglobin) are frequently exposed to toxins (X. Li et al., 2010). The hematological parameters of the treated groups in this study that received a dose higher or equal to 250 mg/kg showed a significant decrease in red blood cells (RBC) and white blood cells (WBC) compared to the control group. The decrease in the number of RBC after administering a product may be due to hemolysis and/or inhibition of hematopoiesis caused by the bioactive components of the extract (Wu et al., 2018). Some plant compounds have been known to alter the metabolism of erythrocytes and damage their membrane, leading to hemolysis (Ruiz et al., 2022). Ferutinin, a sesquiterpene compound found in *Ferula communis*, has been shown in previous studies to induce apoptosis in RBC. This process, known as erythroptosis, is characterized by an increase in caspase-3 activity and cytosolic free calcium ion levels, which are believed to be involved in the mechanism by which ferutinin causes RBC apoptosis (Gao et al., 2013). The decrease in white blood cell count may be attributed to the antiproliferative and anti-inflammatory effects of certain bioactive compounds found in *Ferula communis* (Akaberi et al., 2015). The significant decrease in the count of platelets was observed in both sexes, which may be due to several factors, including a direct toxic effect on the bone marrow, where platelets are produced and/or interference with the normal function or lifespan of platelets, leading to their premature destruction or impaired functioning Ruiz et al., 2022). It is important to note that a decrease in platelet count can have serious consequences, as platelets are essential for proper blood clotting. Platelet depletion, also known as thrombocytopenia, can lead to severe bleeding. In fact, previous studies have shown that the toxic variety of *Ferula communis* can cause hemorrhaging in animals due to high levels of prenylated coumarins (Ruiz et al., 2022).

In terms of biochemical parameters, this study found no significant changes in the levels of ALT and AST, which are commonly used to evaluate liver toxicity. This suggests that FC-Ext did not significantly harm the liver. Additionally, creatinine levels, a marker of kidney function, were similar across groups, indicating that FC-Ext did not adversely affect the kidneys. These findings were supported by histological analysis, which did not reveal significant damage or alteration in liver and kidney tissues.

Finally, it is crucial to note that while the present study offers a thorough examination, it is important to recognize and address several notable limitations that should be taken into account. One limitation is the use of a limited number of dosing groups (125 mg/kg, 250 mg/kg, and 500 mg/kg). Although these doses provided insightful information, it is essential to include higher dose levels in subsequent research to ensure a thorough assessment of the extract's safety, specifically concerning hematological parameters. Moreover, our study focused on the short-term subacute toxicity over 28 days, while extended durations would allow for assessment of long-term effects and potential cumulative impacts. Comprehensive toxicity evaluation should also explore other organ systems and toxicological endpoints such as neurotoxicity, reprotoxicity, and genotoxicity. Further research addressing these limitations will enhance our understanding of the extract's safety profile for informed judgment regarding its use.

## 5 Conclusion

The acute and subacute toxicity of *Ferula communis* aqueous extract was evaluated in this study. The results indicate that the extract has low acute toxicity based on LD_50_ values for both oral and intraperitoneal routes. While no severe toxic effects on vital organs were observed during the 28-day subacute toxicity test, caution is warranted due to the significant decrease in red and white blood cell counts, as well as platelets, potentially leading to hemolysis, apoptosis, and impaired clotting. Further research involving higher doses, different administration routes, and longer durations is necessary to validate these findings. Additionally, comprehensive assessments of neurotoxicity, reprotoxicity, genotoxicity, and the toxicity of bioactive constituents should be conducted. The potential risks associated with blood parameters must be considered before utilizing *Ferula communis* extract.

## Declaration of Competing Interest

The authors declare that they have no known competing financial interests or personal relationships that could have appeared to influence the work reported in this paper.
